# Efficacy and safety of corticosteroid and immunosuppressive agent combination therapy for pediatric IgA nephropathy: a meta-analysis

**DOI:** 10.3389/fped.2025.1709402

**Published:** 2026-01-15

**Authors:** Xilin Xiong

**Affiliations:** Pediatric Department, The First Affiliated Hospital of Jinan University, Guangzhou, Guangdong, China

**Keywords:** children, corticosteroids, IgA nephropathy, immunosuppressive agents, meta-analysis

## Abstract

**Background:**

IgA nephropathy (IgAN) is a common primary glomerular disease in children. The efficacy and safety of glucocorticoid (GC) and immunosuppressive therapies remain debated. This study aimed to evaluate their clinical effectiveness and safety in pediatric IgAN.

**Methods:**

A systematic search was conducted in six databases (PubMed, Web of Science, Cochrane, Embase, CNKI, Wanfang, and VIP), yielding 404 studies, of which eight met inclusion criteria. Eligible studies included RCTs or retrospective studies involving pediatric patients (≤18 years) treated with immunosuppressants plus GC. Outcomes assessed included proteinuria, hematuria, serum creatinine, and adverse events. Two researchers independently extracted and analyzed data. Network meta-analysis (NMA) was performed using R software, incorporating network plots, forest plots, and SUCRA rankings.

**Results:**

Network topology showed strong links between GC monotherapy and regimens such as “tacrolimus + GC” and “mycophenolate mofetil (MMF) + GC.” SUCRA rankings indicated superior efficacy of combination therapies in reducing proteinuria and hematuria. Forest plots revealed that all combination regimens significantly reduced proteinuria compared to GC alone (*P* < 0.05), with only MMF + GC significantly improving hematuria (*P* < 0.05). No significant differences in adverse event rates were found among treatment groups (*P* > 0.05). Funnel plots suggested minimal publication bias.

**Conclusion:**

Combination therapies, especially tacrolimus + GC and MMF + GC, offer greater efficacy than GC monotherapy in pediatric IgAN, without increasing adverse events. These findings support their clinical application, though larger studies are needed to validate results and optimize treatment strategies.

## 1 Introduction

Immunoglobulin A nephropathy (IgAN) is one of the most common primary glomerular diseases worldwide, particularly prevalent in adolescents and school-age children (1). Early studies primarily linked IgAN with hematuria following respiratory tract infections (2), but subsequent research has revealed a more diverse clinical presentation, including episodic hematuria, persistent hematuria, proteinuria, edema, and in some cases, progression to end-stage renal disease (ESRD) (3). Early diagnosis and intervention are essential to improve clinical outcomes in patients with IgAN. However, the pathogenesis of IgAN is multifactorial and not yet fully elucidated. Current understanding is often conceptualized within the “multi-hit” model, which involves the production of poorly galactosylated IgA1 (hit 1), the formation of autoantibodies against these abnormal IgA1 molecules (hit 2), and the subsequent formation and glomerular deposition of immune complexes leading to inflammation and injury (hit 3) (4, 5). Despite these advances, a universally effective and specific treatment targeting the underlying pathogenesis has not been established. According to the 2025 KDIGO guidelines, the management of IgAN mainly focuses on optimized supportive therapy, including blood pressure control, proteinuria reduction, glucocorticoid (GC), and immunosuppressive agents (6). However, high-quality evidence for pediatric IgAN treatment remains scarce, and although the KDIGO guidelines suggest that pediatric management could follow adult protocols, this approach lacks comprehensive validation for children (7).

Although GC and immunosuppressive agents are widely used in the treatment of IgAN, their efficacy and safety in pediatric populations remain inconclusive. GC, as a core treatment for IgAN, are thought to significantly reduce proteinuria and hematuria, offering some protection to kidney function and delaying ESRD (8, 9). However, in the pediatric population, prolonged or high-dose GC therapy is associated with distinct adverse effects, including growth suppression, weight gain, and disturbances in bone metabolism, which are of particular concern in developing children (10). Tacrolimus, an immunosuppressive agent that inhibits calcineurin, has been suggested to provide renal protection in IgAN patients, although the risk of adverse events remains increased (11). While some studies have explored the efficacy and safety of GC or immunosuppressive agents alone, there is a lack of systematic evaluations of their combined use in pediatric IgAN.

In recent years, the combination of GC and immunosuppressive agents has become a clinical trend (12). However, research specifically targeting pediatric patients remains limited and varies in quality. Meta-analysis, as a method for integrating findings from multiple studies, can provide robust evidence to inform clinical decision-making by synthesizing diverse research outcomes. This study aims to systematically evaluate the efficacy and safety of GC combined with immunosuppressive agents in pediatric IgAN through a network meta-analysis. Key outcomes include proteinuria levels, hematuria levels, and the incidence of adverse events. By comprehensively analyzing existing randomized controlled trials (RCTs) and retrospective studies, this study seeks to clarify the therapeutic value of this approach in managing pediatric IgAN.

## 2 Materials and methods

### 2.1 Literature search

This study conducted a systematic literature search to evaluate the efficacy and safety of immunosuppressive agents combined with glucocorticoids in pediatric patients with IgA nephropathy. Comprehensive searches were performed in six major databases: PubMed, Web of Science, Cochrane, Embase, China National Knowledge Infrastructure (CNKI), Wanfang, and VIP. Search terms included “child”, “adolescent”, “pediatric”, “youth”, “childhood”, “IgA nephropathy”, “Berger disease”, “glomerulonephritis”, “IgA”, “Tacrolimus”, “Mycophenolate Mofetil”, “Cyclophosphamide”, “Cyclosporine A”, “Azathioprine”, and “Glucocorticoids”. The number of studies retrieved varied across databases, ranging from 4 in the VIP database to 291 in Embase, with a total of 404 studies identified. After deduplication and screening, 8 studies meeting the inclusion criteria were selected for final analysis. All searches were independently conducted by two researchers, and studies were screened based on predefined inclusion and exclusion criteria.

### 2.2 Inclusion and exclusion criteria

#### 2.2.1 Inclusion criteria

Studies must meet all of the following conditions: (1) Study Population: Pediatric patients aged ≤18 years with IgA nephropathy diagnosed by renal biopsy. Patients must have documented proteinuria [defined as 24-hour urinary protein excretion ≥0.5 g/d or urine protein-to-creatinine ratio (UPCR) ≥ 0.5 g/g] and/or hematuria [urinary red blood cell count ≥5/high-power field (HPF)]. (2) Intervention: The treatment group must receive a combination therapy of GC and immunosuppressive agents (such as mycophenolate mofetil, azathioprine, cyclophosphamide, tacrolimus, etc.). (3) Study Design: Randomized controlled trials (RCTs) or retrospective studies. (4) Outcomes: Report at least one of the following: Primary outcomes—complete remission rate or total effective rate. Secondary outcomes—improvement in proteinuria levels, improvement in hematuria levels (e.g., reduction in urinary red blood cell count), renal function improvement (e.g., eGFR or serum creatinine), and incidence of adverse events.

#### 2.2.2 Exclusion criteria

Exclusion of studies with: (1) Participants with secondary IgA nephropathy (such as Henoch-Schönlein purpura nephritis, hepatitis B-related nephropathy). (2) Non-randomized controlled studies (e.g., observational studies, case reports, conference abstracts). (3) Incomplete or non-extractable data. (4) Unclear definitions of outcome measures.

### 2.3 Study selection and data extraction

**Figure 1:**
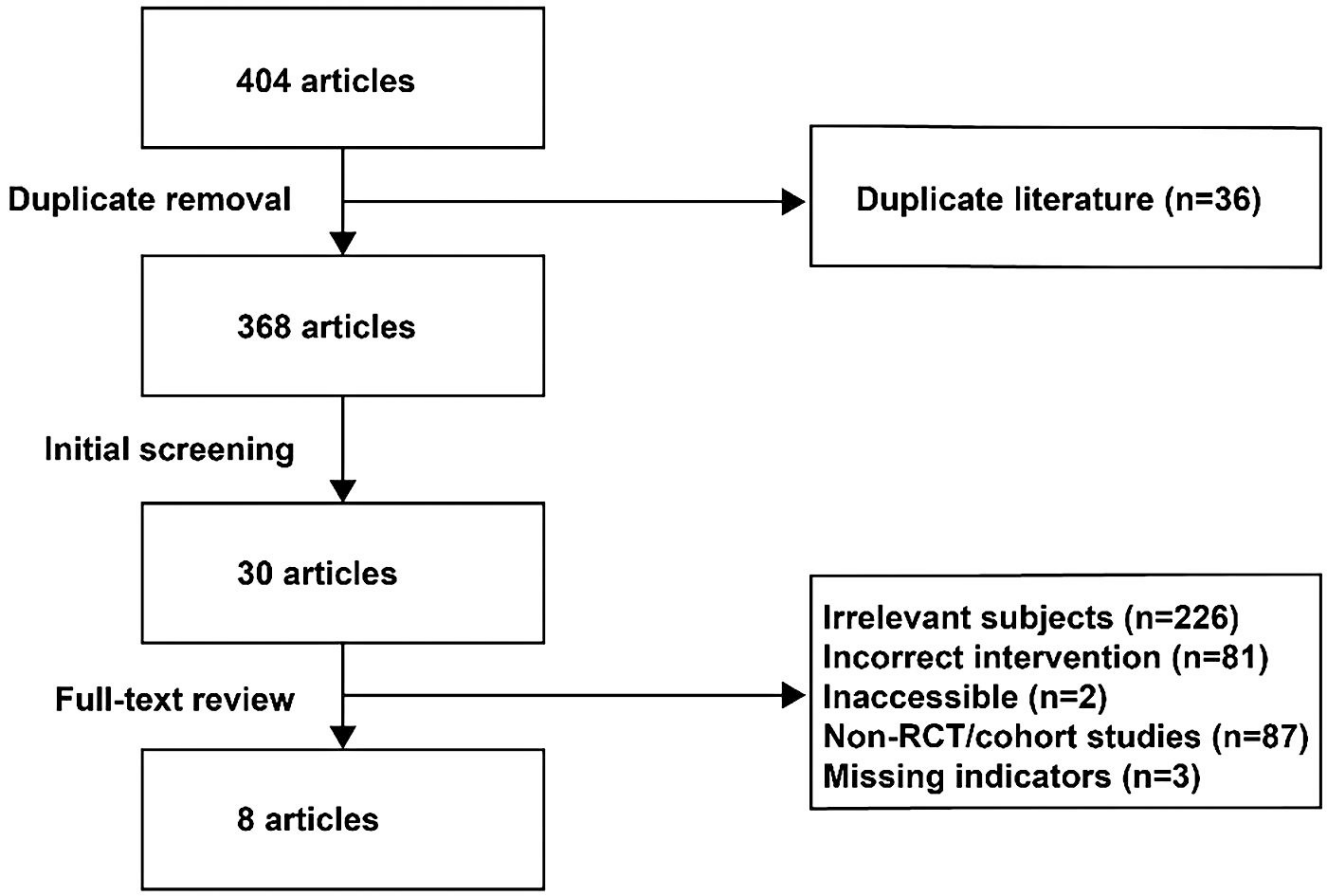
Study selection process.

Two researchers independently screened titles and abstracts to exclude irrelevant studies. Full texts of potentially eligible studies were reviewed to confirm inclusion. The study selection process is illustrated in Figure 1. Data extraction was independently performed by two researchers using standardized forms to record relevant details, including basic study information (e.g., first author, publication year, country, study type), sample size, interventions, proteinuria levels, hematuria levels, and adverse event incidence. For studies reporting outcomes at multiple time points, data from the longest follow-up were prioritized. Discrepancies in data extraction were resolved through discussion or adjudication by a third researcher.

### 2.4 Network meta-analysis methods

#### 2.4.1 Network plot construction

A network topology plot was constructed using the “netmeta” package (version 2.9.0) in R software. Nodes represented different interventions, while edge thickness indicated the frequency of comparisons and sample sizes. The plot provided a visual overview of direct and indirect comparisons, ensuring the network structure's completeness and connectivity.

#### 2.4.2 Treatment ranking assessment

Treatment ranking was assessed using the Surface Under the Cumulative Ranking Curve (SUCRA) method. SUCRA values, ranging from 0% to 100%, were calculated by integrating cumulative probabilities for each treatment. Higher SUCRA values indicated superior efficacy and safety. SUCRA calculations were performed using the “netmeta” package in R software.

#### 2.4.3 Forest plot analysis

Forest plots were utilized to present effect estimates from direct, indirect, and network evidence in the network meta-analysis (NMA). These plots displayed the distribution of effect sizes and their confidence intervals, enabling assessment of relative efficacy, statistical significance, and uncertainty. Forest plots were generated using R software and depicted standardized mean differences (SMDs) or risk ratios (RRs). Effect sizes for individual studies were represented by squares proportional to study weight, with horizontal lines denoting 95% confidence intervals.

#### 2.4.4 Heterogeneity testing and model selection

Heterogeneity was evaluated using Cochran's *Q* test and the *I*^2^ statistic. The I² values quantified inter-study heterogeneity: *I*^2^ < 25% indicated low heterogeneity, 25% ≤ *I*^2^ ≤ 50% indicated moderate heterogeneity, and *I*^2^ > 50% indicated high heterogeneity. Fixed-effects models were applied for low heterogeneity, while random-effects models were used for high heterogeneity to obtain more accurate effect estimates.

#### 2.4.5 Funnel plot analysis

Publication bias and small-study effects were assessed using funnel plots. Funnel plot symmetry was examined, and asymmetry suggested potential biases, warranting further evaluation using Egger's test. Funnel plots were created using R software.

### 2.5 Statistical analysis

All statistical analyses were performed using R software version 4.2.1 (Lucent Technologies Inc., Union, NJ, USA, https://www.r-project.org/). Continuous outcomes (e.g., proteinuria and hematuria levels) were analyzed as standardized mean differences (SMDs), while adverse event incidence was analyzed as risk ratios (RRs). Network meta-analysis was implemented using the “netmeta” package (version 2.9.0). Sensitivity and subgroup analyses were conducted to explore sources of heterogeneity and assess the robustness of findings.

## 3 Results

### 3.1 Literature retrieval results and basic characteristics of included studies

This study systematically searched multiple databases, including PubMed, Web of Science, Cochrane, Embase, China National Knowledge Infrastructure (CNKI), Wanfang, and VIP, yielding 404 relevant articles After screening, a total of 4 RCTs and 4 retrospective studies were included (13–20), involving 517 pediatric patients (Table 1). The included studies were published between 2004 and 2023, with four studies conducted in Japan and four in China. Sample sizes ranged from 22 to 96 patients. The interventions evaluated encompassed GC monotherapy and combination therapies of GC with various immunosuppressive agents, including tacrolimus, mycophenolate mofetilMMF, mizoribine, cyclophosphamide, and azathioprine. The most frequently compared regimens were “Tacrolimus + GC” vs. “mycophenolate mofetil + GC” (3 studies) and “Mizoribine + GC” vs. GC monotherapy (3 studies). All studies reported outcomes on urinary protein levels, hematuria levels, and the incidence of adverse events; most also reported complete/total remission rates and renal function indicators. The included studies evaluated the efficacy of various treatment regimens, including direct comparisons of different immunosuppressants combined with GC vs. the use of GC alone in treating pediatric IgA nephropathy, as well as comparisons between different combination therapies. All studies reported clinical outcomes on urinary protein levels, hematuria levels, and the incidence of adverse events.

**Table 1:** Basic information of included studies.

Author	Year	Study type	Region	Total	Group1	Group2	Group3	Clinical outcome
Kawasaki et al. (13)	2004	Retrospective study	Japan	61	Glucocorticoid	Glucocorticoid	Mizoribine + Glucocorticoid	②③④⑤
Ikezumi et al. (14)	2023	Retrospective study	Japan	90	Glucocorticoid	Mizoribine + Glucocorticoid	NA	①②③④⑤
Yoshikawa et al. (15)	2006	RCT	Japan	83	Azathioprine + Glucocorticoid	Glucocorticoid	NA	②③④
Zhang et al. (16)	2022	RCT	China	46	Glucocorticoid	Mizoribine + Glucocorticoid	NA	①②④⑤
Hu (17)	2017	RCT	China	22	Mycophenolate mofetil + Glucocorticoid	Cyclophosphamide + Glucocorticoid	Glucocorticoid	②③④⑤
Luo, et al. (18)	2022	RCT	China	96	Tacrolimus + Glucocorticoid	Mycophenolate mofetil + Glucocorticoid	NA	①②③④⑤
Wu et al. (19)	2020	Retrospective study	China	76	Tacrolimus + Glucocorticoid	Mycophenolate mofetil + Glucocorticoid	NA	①②③④⑤
Zhang et al. (20)	2019	Retrospective study	China	43	Tacrolimus + Glucocorticoid	Mycophenolate mofetil + Glucocorticoid	NA	①②③④⑤

① Complete remission rate or total effective rate; ② Proteinuria levels; ③ hematuria levels; ④ Function; ⑤ Incidence of adverse events.

### 3.2 Network topology analysis

**Figure 2:**
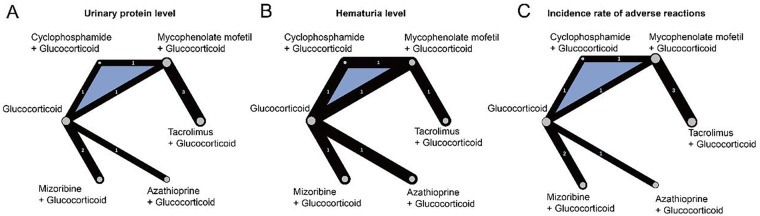
Network topology diagram. **(A)** Network analysis of the effect of interventions on urinary protein levels. **(B)** Network analysis of the effect of interventions on hematuria levels. **(C)** Network analysis of the effect of interventions on adverse event incidence.

The network topology diagram illustrates the direct and indirect comparative relationships between different treatment regimens. Nodes represent the various treatment strategies, while the lines connecting them represent direct comparisons from the studies. This provides a clear visualization of the evidence network structure and its complexity. The analysis indicated that there is direct or indirect evidence supporting the comparisons between different treatments, consistent with the basic requirements of a network meta-analysis (NMA). Notably, the connection between “Tacrolimus + GC” and “Mycophenolate mofetil + GC” with GCs alone was relatively tight, suggesting that these combinations may be more effective than GC monotherapy. Additionally, there was a three-arm relationship involving GC, Cyclophosphamide + GC, and Mycophenolate mofetil + GC (Figure 2).

### 3.3 Assessment of treatment priorities

**Figure 3:**
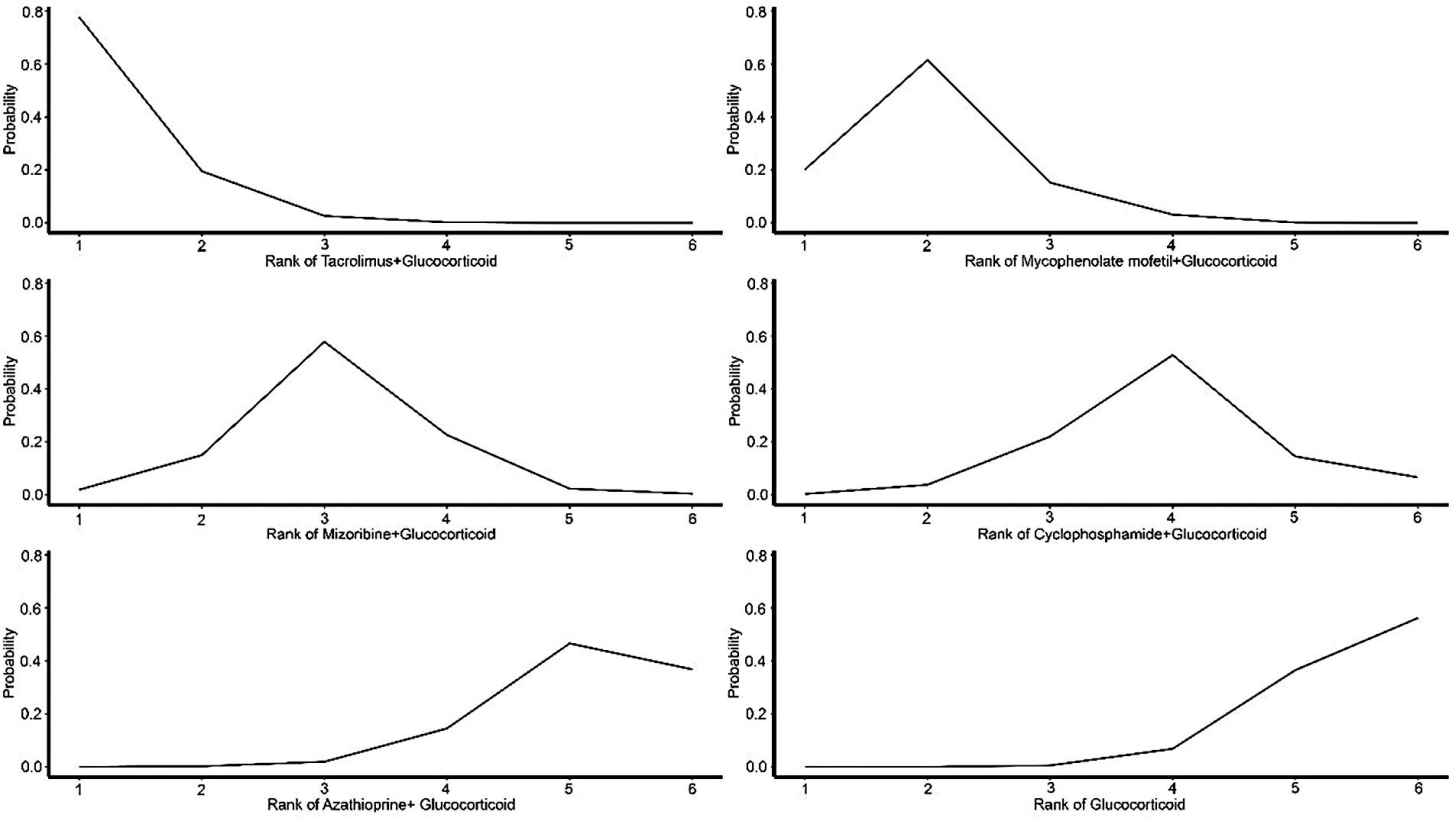
Probability ranking of treatments for urinary protein.

**Figure 4:**
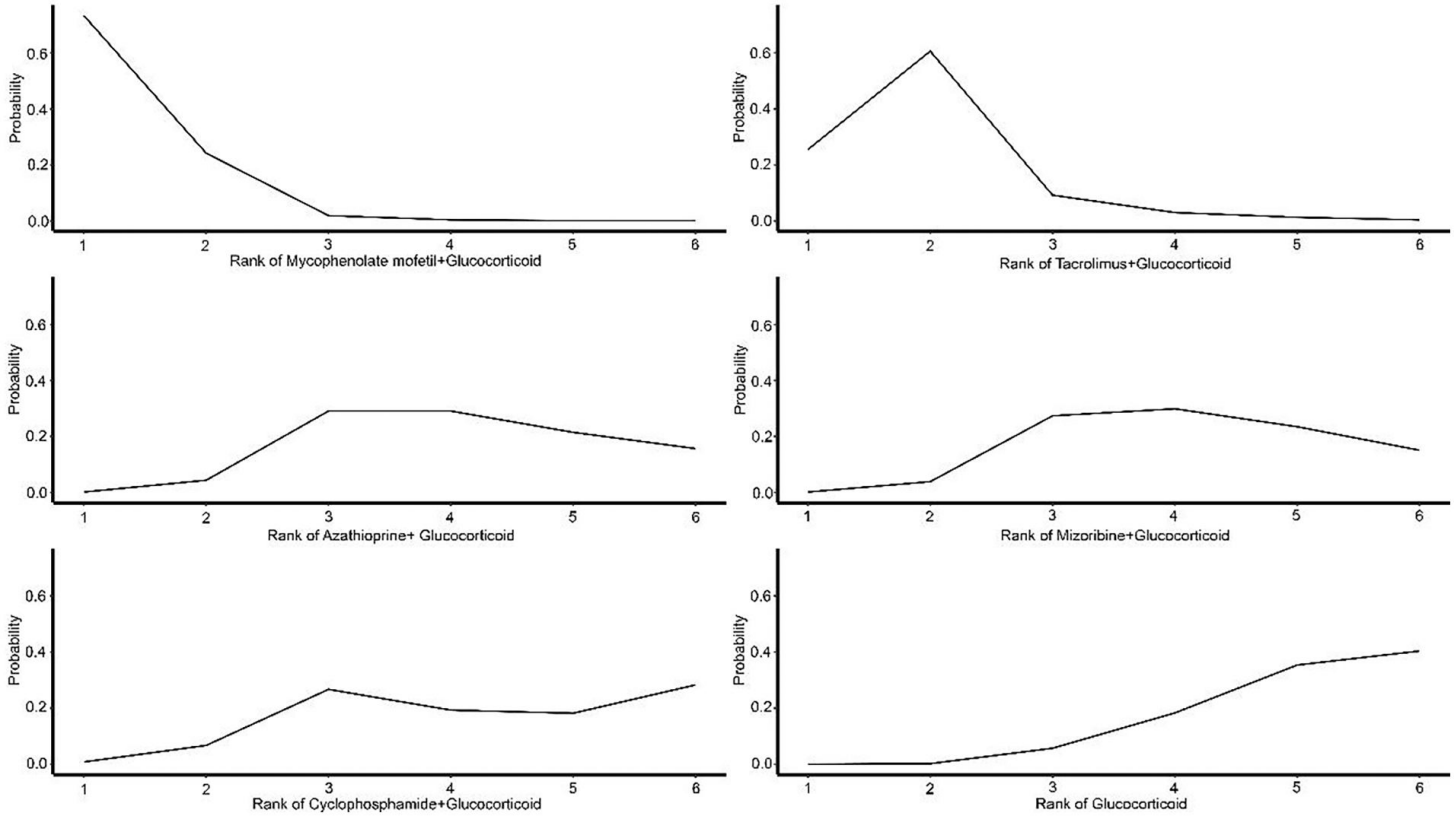
Probability ranking of treatments for hematuria.

**Figure 5:**
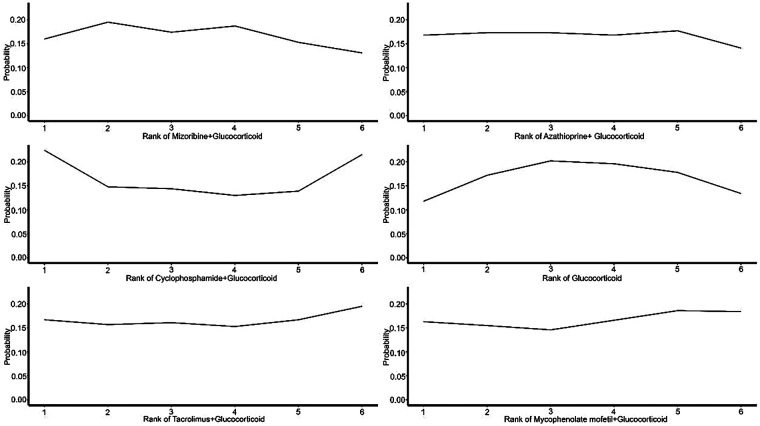
Probability ranking of treatments for adverse events.

The comprehensive clinical value and relative priority of each intervention were ranked using the Surface Under the Cumulative Ranking Curve (SUCRA). Higher-ranked treatment options showed more significant efficacy. The analysis revealed that all combination interventions ranked better than GC monotherapy in improving urinary protein and hematuria levels, with “Tacrolimus + GC” and “Mycophenolate mofetil + GC” leading the rankings, showing more substantial treatment effects. This indicates that “Tacrolimus + GC” and “Mycophenolate mofetil + GC” are significantly more effective than other treatment regimens in improving urinary protein and hematuria levels (Figures 3–5).

### 3.4 Efficacy analysis relative to GCs

**Figure 6:**
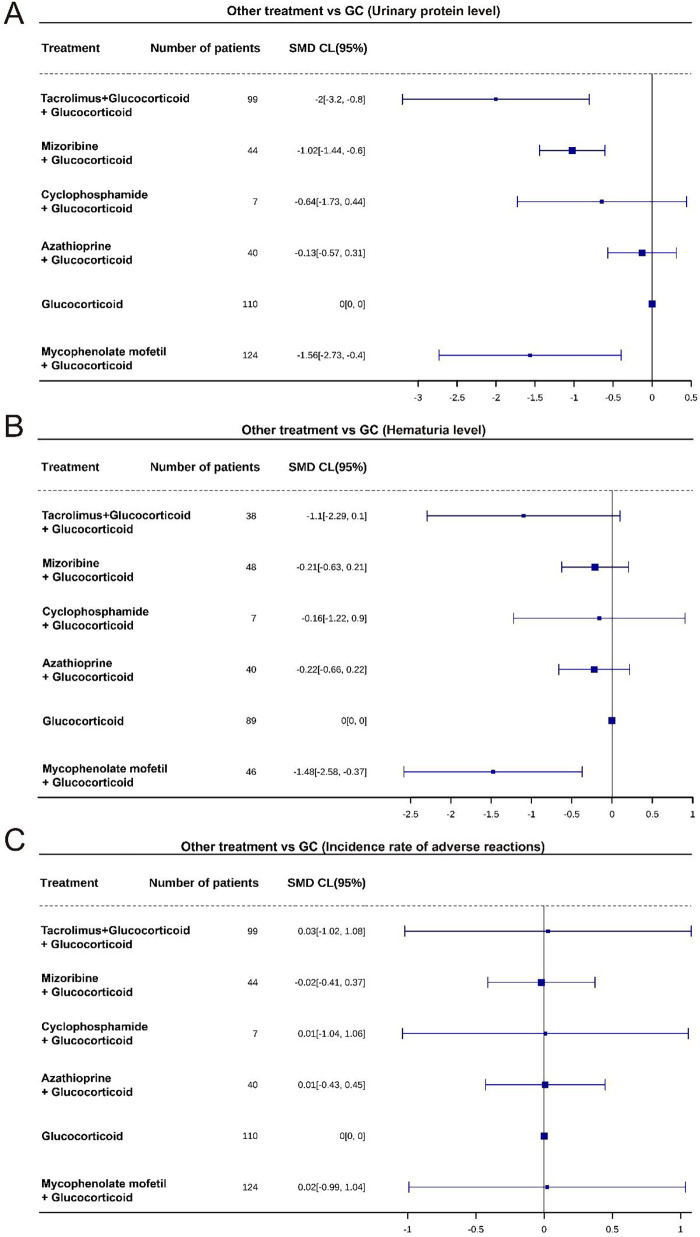
Comparison results between different treatments and the reference (GC monotherapy). **(A)** Forest plot of urinary protein efficacy relative to GC. **(B)** Forest plot of hematuria efficacy relative to GC. **(C)** Forest plot of adverse event efficacy relative to GC.

In the analysis of urinary protein levels, all combination treatments (Tacrolimus + GC, Mizoribine + GC, Mycophenolate mofetil + GC) significantly alleviated urinary protein levels and demonstrated better effects compared to GC monotherapy (*P* < 0.05) (Figure 6, Table 2). Regarding hematuria levels, only Mycophenolate mofetil + GC significantly improved hematuria levels (*P* < 0.05) (Figure 6, Table 3). However, in the analysis of adverse event incidence, no significant differences were observed between the combination treatments and GC monotherapy (*P* > 0.05), indicating that the safety profiles of the different combination therapies are similar (Figure 6, Table 4).

**Table 2:** Efficacy analysis of urinary protein levels relative to GC.

Treatment	SMD	SMD_se	lower_ci	upper_ci	statistic	*P*
Tacrolimus + Glucocorticoid	−2.00	0.61	−3.20	−0.80	−3.28	0.001
Mizoribine + Glucocorticoid	−1.02	0.21	−1.44	−0.60	−4.78	<0.001
Cyclophosphamide + Glucocorticoid	−0.64	0.55	−1.73	0.44	−1.17	0.244
Azathioprine + Glucocorticoid	−0.13	0.22	−0.57	0.31	−0.58	0.565
Glucocorticoid	0.00	0.00	0.00	0.00		
Mycophenolate mofetil + Glucocorticoid	−1.56	0.60	−2.73	−0.40	−2.63	0.009

**Table 3:** Efficacy analysis of hematuria levels relative to GC.

Treatment	SMD	SMD_se	lower_ci	upper_ci	statistic	*P*
Tacrolimus + Glucocorticoid	−1.10	0.61	−2.29	0.10	−1.80	0.07
Mizoribine + Glucocorticoid	−0.21	0.21	−0.63	0.21	−0.99	0.32
Cyclophosphamide + Glucocorticoid	−0.16	0.54	−1.22	0.90	−0.29	0.77
Azathioprine + Glucocorticoid	−0.22	0.22	−0.66	0.22	−0.98	0.32
Glucocorticoid	0.00	0.00	0.00	0.00		
Mycophenolate mofetil + Glucocorticoid	−1.48	0.56	−2.58	−0.37	−2.61	0.01

**Table 4:** Efficacy analysis of adverse event incidence relative to GC.

Treatment	SMD	SMD_se	lower_ci	upper_ci	statistic	*P*
Tacrolimus + Glucocorticoid	0.03	0.54	−1.02	1.08	0.05	0.96
Mizoribine + Glucocorticoid	−0.02	0.20	−0.41	0.37	−0.10	0.92
Cyclophosphamide + Glucocorticoid	0.01	0.53	−1.04	1.06	0.02	0.99
Azathioprine + Glucocorticoid	0.01	0.22	−0.43	0.45	0.04	0.97
Glucocorticoid	0.00	0.00	0.00	0.00		
Mycophenolate mofetil + Glucocorticoid	0.02	0.52	−0.99	1.04	0.04	0.97

### 3.5 Efficacy analysis of various treatments

**Figure 7:**
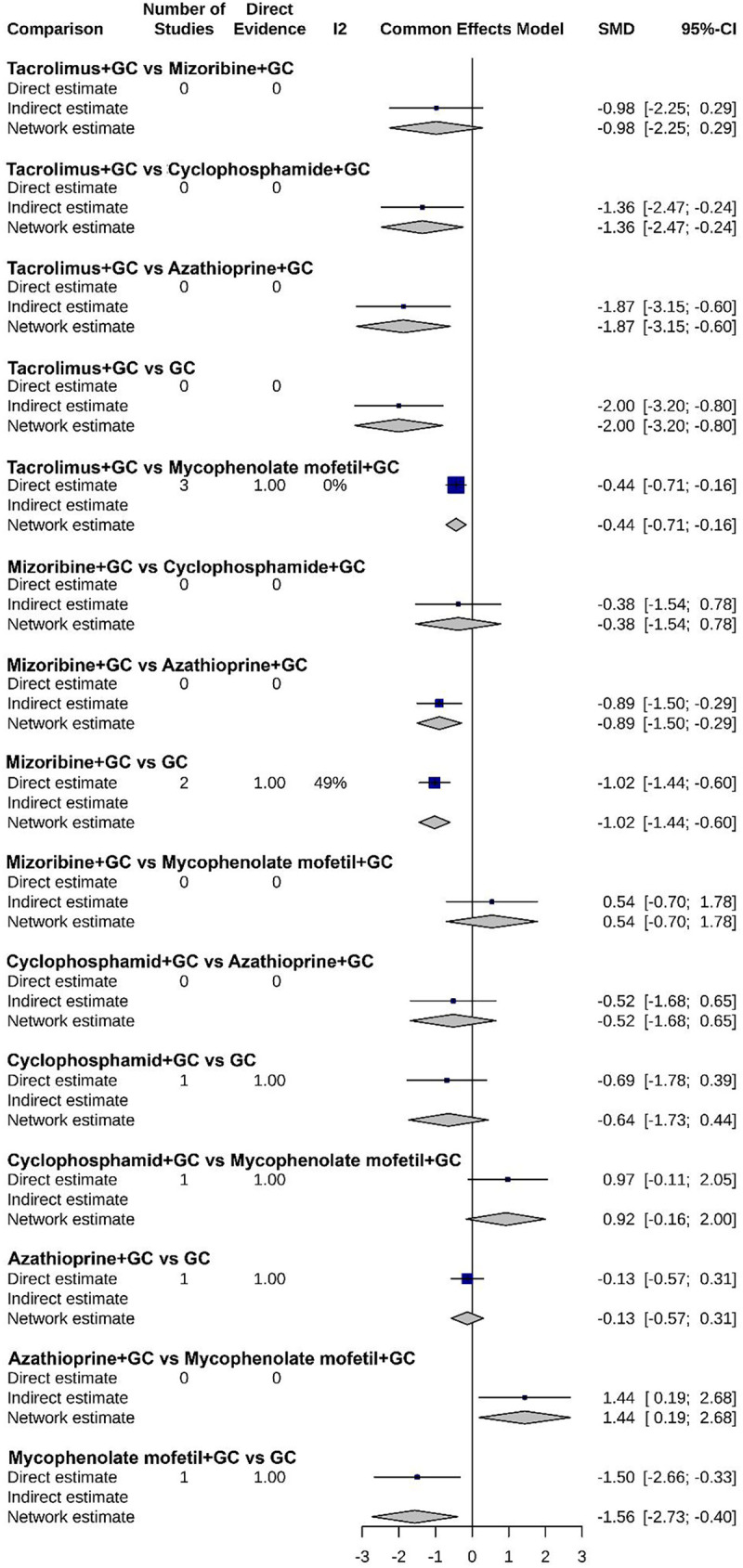
Forest plot of the relative effect on urinary protein levels for each treatment regimen.

**Figure 8:**
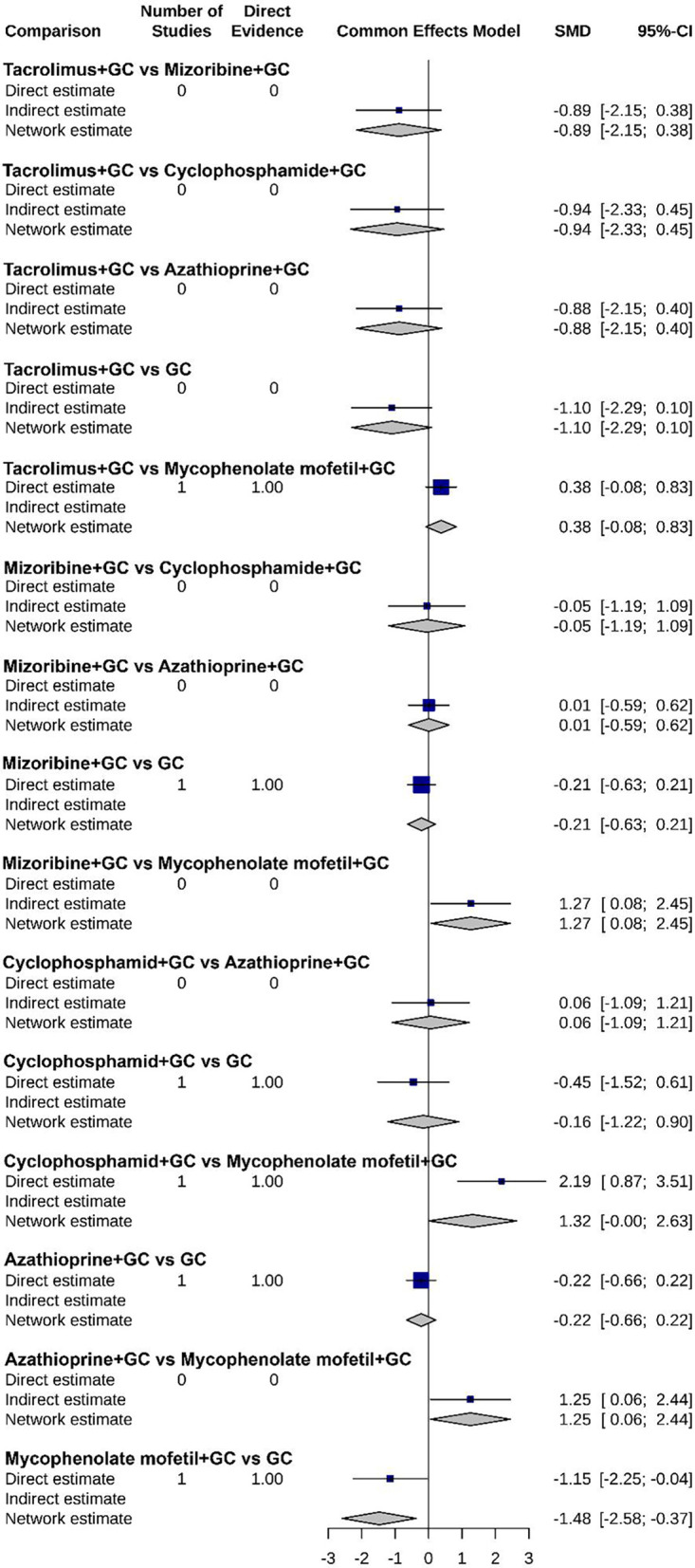
Forest plot of the relative effect on hematuria levels for each treatment regimen.

**Figure 9:**
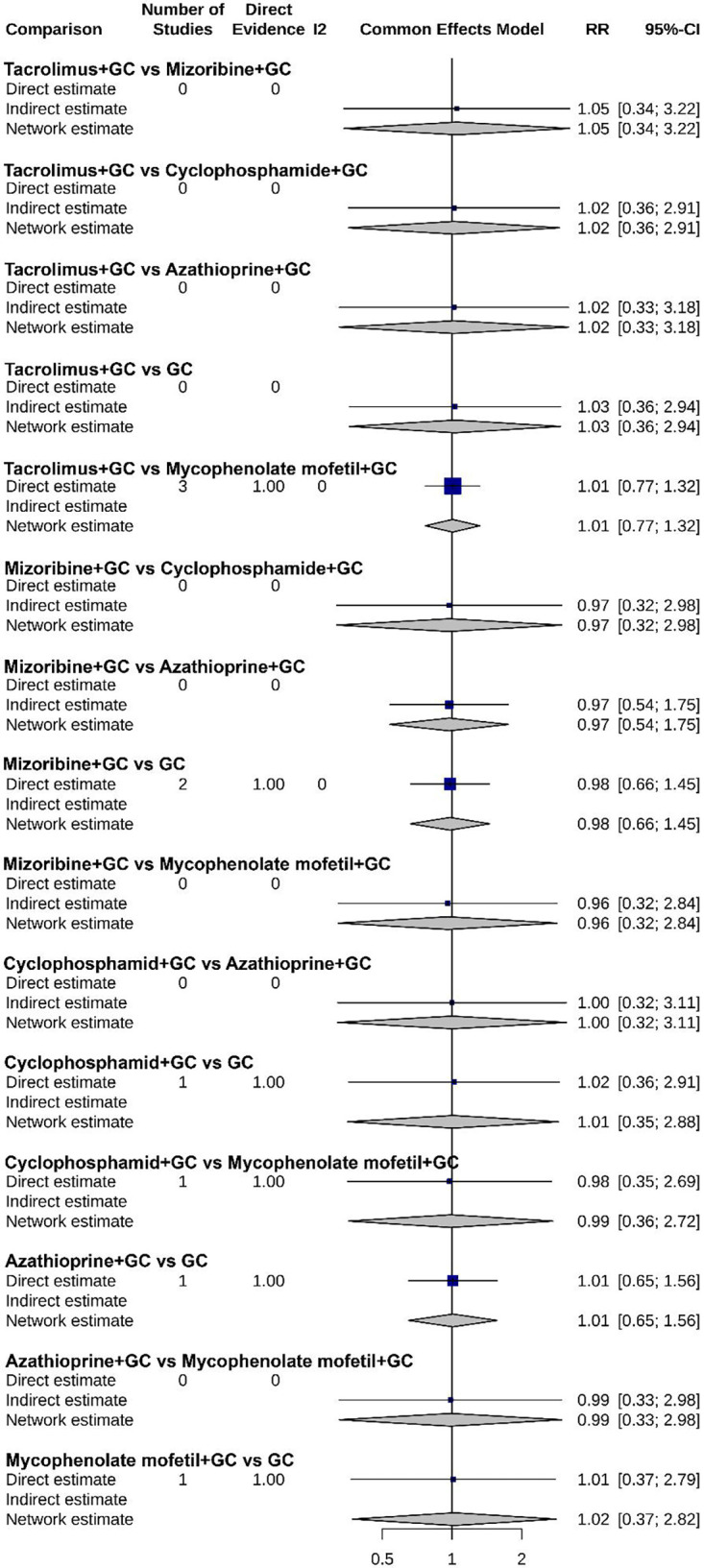
Forest plot of the relative effect on adverse event incidence for each treatment regimen.

As shown in Figures 7–9, the forest plots provide a visual representation of the relative effects of different treatments on urinary protein, hematuria, and adverse event incidence. The comprehensive analysis indicated that “Tacrolimus + GC” exhibited superior efficacy over other regimens, including “Mizoribine + GC”, “Cyclophosphamide + GC”, “Azathioprine + GC”, and “GC monotherapy”, in terms of both urinary protein and hematuria levels. For urinary protein, Mycophenolate mofetil + GC also showed significantly better results than other combination therapies. It is noteworthy that no significant differences were found among the treatment regimens regarding adverse event incidence, suggesting that all treatments have similar safety profiles.

### 3.6 Safety profile analysis

The safety of combination therapies was evaluated based on the incidence and types of adverse events reported in the included studies. No significant differences in the overall adverse event rate were observed between any combination therapy group and GC monotherapy (*P* > 0.05), indicating that the addition of immunosuppressive agents did not increase safety risks. Specific adverse events varied by the type of immunosuppressant: ① Tacrolimus + GC: Adverse events mainly included transient blood glucose elevation, mild renal tubular function impairment, and gastrointestinal discomfort. For example, in the study by Wu et al. (19), 2 out of 38 patients in the tacrolimus group developed elevated blood glucose, which resolved with symptomatic management; 1 case of tubular function impairment was reported and reversed after adjusting tacrolimus blood concentration to the target range (5–10 μg/L). ② Mycophenolate mofetil (MMF) + GC: The most common adverse events were infections (predominantly upper respiratory tract infections) and mild liver function abnormalities. Luo et al. (18) noted a 20.00% adverse event rate in the MMF group, including 3 cases of infection and 1 case of transaminase elevation, which improved with anti-infective treatment or dose adjustment. ③ Mizoribine + GC: Adverse events were mainly respiratory tract infections and transient hyperuricemia. Zhang et al. (16) reported that 54.17% of patients in the mizoribine group experienced respiratory tract infections, but the recurrence rate of IgAN (12.50%) was significantly lower than that in the GC monotherapy group (50.00%); 2 cases of hyperuricemia were controlled with allopurinol without treatment interruption. ④ Azathioprine + GC: Leukopenia and elevated transaminase were the main adverse events. Yoshikawa et al. (15) documented 4 cases of leukopenia and 2 cases of transaminase elevation in the azathioprine combination group, which resolved after temporary drug withdrawal or dose reduction. ⑤ Cyclophosphamide + GC: Isolated cases of liver function abnormalities were reported. Hu (17) observed 1 case of elevated alanine transaminase in the cyclophosphamide group, which recovered spontaneously within 2 months without specific treatment. All adverse events were mild to moderate and manageable with symptomatic treatment, dose adjustment, or temporary drug withdrawal, with no severe or life-threatening events reported across the included studies.

### 3.7 Funnel plot analysis

**Figure 10:**
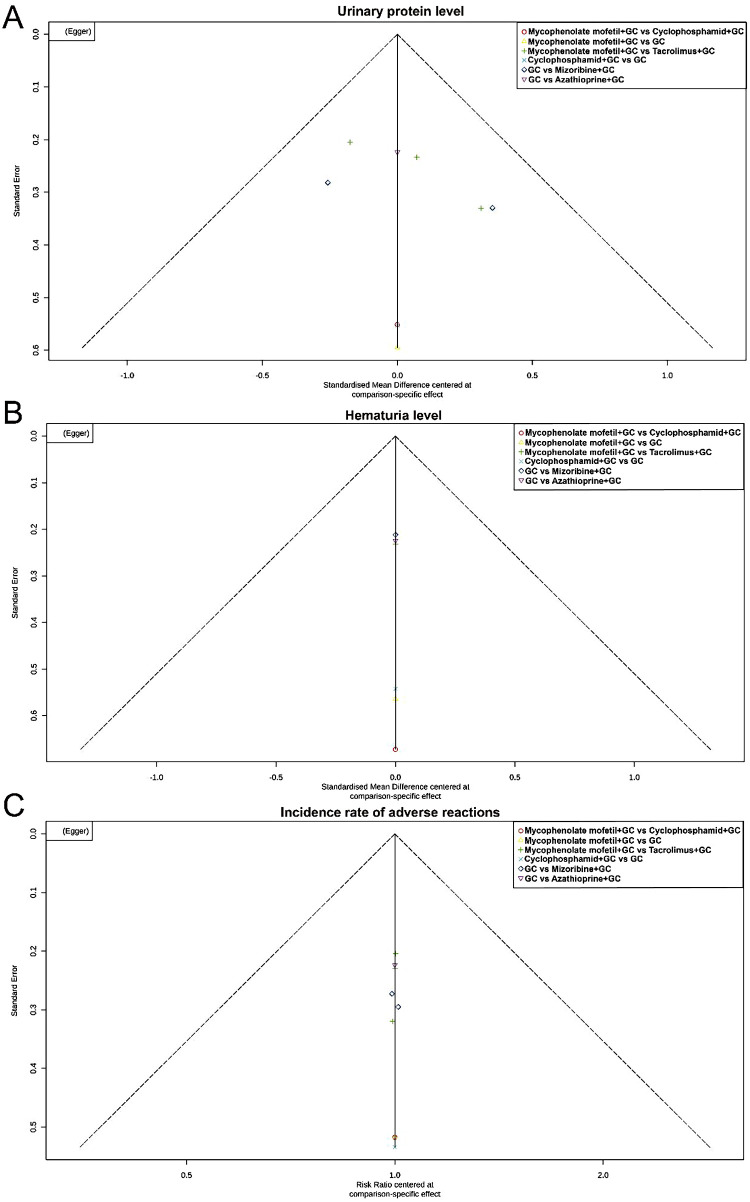
Funnel plots. **(A)** Funnel plot for urinary protein levels. **(B)** Funnel plot for hematuria levels. **(C)** Funnel plot for adverse event incidence.

The funnel plot, which illustrates the relationship between the effect size estimates of each study and their standard errors, helps assess the presence of publication bias. The funnel plot showed symmetric distribution of study results across all three outcome measures, indicating no significant publication bias or small sample effects (Figure 10). Nevertheless, given the limited number of included studies, further research is needed to confirm the robustness of the findings.

## 4 Discussion

IgA nephropathy (IgAN) is a major cause of chronic kidney disease in children, with a complex pathogenesis that includes abnormal activation of the immune system, deposition of IgA1 immune complexes in the glomeruli, and subsequent chronic inflammatory responses (21). Although corticosteroid (GC) therapy can partially alleviate inflammation and reduce proteinuria, its efficacy is often limited, especially in severe or high-risk patients (e.g., those with high baseline proteinuria or impaired renal function) (22). In recent years, more studies have explored the efficacy of combining corticosteroids with immunosuppressive agents, aiming to synergistically reduce inflammation and immune responses, thereby improving long-term outcomes for patients (23, 24). However, the advantages of combination therapy have not been consistently validated across all studies, and research on its applicability in pediatric IgAN is limited. Furthermore, the heterogeneity among studies in patient baseline characteristics, treatment regimens, and outcome definitions has made it difficult to draw definitive conclusions from existing evidence (25). In this context, this study conducted a comprehensive analysis of previous randomized controlled trials (RCTs) to systematically evaluate the efficacy and safety of the combination therapy of glucocorticoids and immunosuppressants in pediatric IgAN. By delving into the sources and potential mechanisms of heterogeneity, new insights were provided for this field.

In the treatment of pediatric IgAN, corticosteroids have long been considered the standard therapeutic approach. However, prolonged reliance on corticosteroids can lead to a range of side effects, such as growth retardation, metabolic disturbances, and osteoporosis (26). Therefore, finding more effective treatments with fewer side effects has become a key goal in clinical practice. Our study found that the combination of immunosuppressive agents with corticosteroids demonstrated significant advantages in improving clinical outcomes. In particular, “Tacrolimus + GC” and “Mycophenolate mofetil + GC” showed much better control of urinary protein and hematuria compared to GC monotherapy. Tacrolimus, a calcineurin inhibitor, effectively suppresses T-cell activation and immune responses (27). Studies have shown that the activity of T-cells is closely associated with renal injury in the immunopathology of IgA nephropathy (28). Research has indicated that tacrolimus treatment in IgAN patients leads to significant improvements in proteinuria and hypoalbuminemia (29). In our meta-analysis, the “Tacrolimus + GC” combination showed the best efficacy in improving urinary protein levels. Mycophenolate mofetil, which inhibits T-cell and B-cell proliferation, reduces immune complex formation and kidney inflammation, playing a crucial role in renal protection (30). This drug has been shown to effectively alleviate proteinuria and reduce recurrence in pediatric IgAN (31). In our analysis, the “Mycophenolate mofetil + GC” combination also demonstrated good efficacy, particularly in controlling urinary protein and improving hematuria.

Regarding safety analysis, although there were no statistically significant differences in adverse event incidence between the various treatment regimens and GC monotherapy, our study found that all combination therapies had a safety profile comparable to that of GC monotherapy. This suggests that combination therapy is safe in pediatric IgAN. Zhang et al. (2018) noted in a retrospective study that patients receiving tacrolimus combined with GC did not experience an increased risk of adverse events and had a relatively high overall safety profile (32). Similarly, Zheng et al. (2023) found that the combination of mycophenolate mofetil and GC was safe in children (33). These studies indicate that while the use of immunosuppressants may increase the risk of certain adverse events, their combination with corticosteroids remains safe, with no significant differences. It is important to note that the incidence of adverse events may vary depending on the type, dosage, and duration of immunosuppressive agents (34), and our study did not clarify these factors. This suggests that future research should more carefully assess the safety profiles of different drugs and treatment regimens.

From a biological mechanism perspective, the advantages of combination therapy may be related to several factors. First, corticosteroids reduce the release of inflammatory factors, thereby alleviating acute inflammatory responses in the glomeruli (35), while immunosuppressive agents inhibit the abnormal activation of T-cells and B-cells, fundamentally reducing the production and deposition of IgA1 immune complexes (36, 37). Second, combination therapy may protect the integrity of the glomerular basement membrane, reducing the occurrence of proteinuria and thereby indirectly alleviating chronic damage to the renal tubulointerstitial tissue (38, 39). These mechanisms work together not only to improve short-term outcomes but also potentially offer long-term renal protection. However, these hypotheses need to be further verified through experimental studies.

Although this study integrated data from existing randomized controlled trials (RCTs) and retrospective studies via network meta-analysis, providing comprehensive evidence for clinical practice, several limitations remain. First, the limited number of included studies, coupled with the presence of multiple treatment subgroups, may reduce statistical power and compromise the robustness and generalizability of the conclusions. Specifically, the sample size of the included studies was relatively small, especially in some treatment groups (e.g., cyclophosphamide + GC), which may affect the generalizability and reliability of the results. SSecond, none of the included studies systematically reported baseline histopathological characteristics using standardized classification systems such as the Oxford MEST-C score. Although all patients were biopsy-confirmed, the lack of detailed and comparable pathological data limits our ability to assess whether treatment effects varied according to initial histological severity. This represents a potential source of heterogeneity and should be addressed in future studies. Third, the number of studies and patients contributing to each treatment node, particularly for some regimens like cyclophosphamide + GC, was limited. This sparsity of data within the network, while allowing for indirect comparisons, mandates cautious interpretation of the relative rankings among all six treatment regimens. The confidence in the point estimates for some pairwise comparisons, especially those relying heavily on indirect evidence, may be constrained. Fourth, a key renal functional outcome, the estimated glomerular filtration rate (eGFR), was not analyzed as a pre-specified outcome in this meta-analysis due to inconsistent reporting across the included studies. The lack of a standardized assessment of eGFR change limits our ability to comprehensively evaluate the impact of these therapies on long-term renal function preservation, which is a critical treatment goal in IgAN. Additionally, although complete remission was initially considered a key efficacy outcome, the variability in its definition across studies prevented its inclusion in the quantitative synthesis. Future studies should adopt standardized criteria for remission to facilitate comparative effectiveness research. Therefore, although network meta-analysis can provide reliable evidence by synthesizing data from multiple studies, further large-scale, high-quality prospective RCTs are needed to validate our conclusions. Additionally, this study only included certain treatment regimens, and other possible combination therapies (e.g., combinations of different immunosuppressants with GC) were not considered. Thus, the applicability of the results may be somewhat limited. Future studies should include a broader range of treatment regimens to further clarify the efficacy and safety of various combination therapies.

In conclusion, our findings suggest that immunosuppressant combinations with corticosteroids offer significant advantages over corticosteroid monotherapy in improving clinical outcomes in pediatric IgAN, particularly in controlling urinary protein and hematuria. In clinical practice, physicians can select the appropriate immunosuppressant combination with corticosteroids based on individual patient conditions. However, since there is currently no unified standard for evaluating treatment efficacy, future research should further explore the long-term effects, recurrence rates, and quality of life of patients. Future studies should focus on the following areas: (1) expanding sample sizes to enhance the credibility of data; (2) conducting multi-center, large-scale prospective RCTs to further confirm the efficacy and safety of combination therapies; (3) evaluating the cost-effectiveness of different combination therapies to help clinicians make more scientifically informed treatment decisions based on economic considerations.

Based on the findings of this meta-analysis, we propose a preliminary treatment algorithm for pediatric IgA nephropathy. For children with active disease (proteinuria ≥0.5 g/d or UPCR ≥0.5 g/g), initial therapy with glucocorticoids (GC) is recommended. If inadequate response is observed after 3–6 months, or in cases of high-risk features (e.g., persistent heavy proteinuria, declining eGFR), combination therapy with an immunosuppressive agent should be considered. Tacrolimus + GC or Mycophenolate mofetil (MMF) + GC may be prioritized given their superior efficacy in reducing proteinuria and hematuria. Regular monitoring of renal function, proteinuria, hematuria, and drug-specific adverse effects is essential throughout treatment. This algorithm should be adapted based on individual patient characteristics, regional availability of drugs, and clinician expertise.

Regarding the mitigation of corticosteroid-related adverse effects, particularly osteoporosis in growing children, we recommend the following preventive strategies: (1) Use the lowest effective dose of glucocorticoids for the shortest duration possible; (2) Ensure adequate calcium and vitamin D supplementation; (3) Encourage weight-bearing physical activity; (4) Consider bone density monitoring in patients on long-term or high-dose steroid therapy; (5) In selected cases, discuss the potential role of bisphosphonates under specialist guidance, though evidence in children remains limited.

Finally, we acknowledge the clinical and pathological continuum between IgA vasculitis (IgAV, formerly Henoch–Schönlein purpura) and IgA nephropathy. A recent comprehensive review by Castañeda et al. (40) underscores that therapeutic strategies for IgA-mediated diseases often overlap across age groups. Given that adults with IgAV experience more frequent and severe nephritis—with glucocorticoids as first-line therapy and emerging evidence supporting biologics such as rituximab—insights from adult IgAV management may offer valuable perspectives for refining immunosuppressive approaches and integrated care in pediatric patients with IgA-related renal disease.

## 5 Conclusion

In summary, this study demonstrates that immunosuppressant combination therapy with corticosteroids is superior to corticosteroid monotherapy in improving urinary protein and hematuria levels in pediatric IgAN, with similar safety profiles. These findings provide new options for clinical treatment of pediatric IgAN and offer directions for future research. However, further high-quality randomized controlled trials and long-term follow-up studies are necessary to validate the long-term efficacy and safety of these treatment regimens.

## Data Availability

The original contributions presented in the study are included in the article/Supplementary Material, further inquiries can be directed to the corresponding author.
